# Correction: Does cultural identity catalyze behavior? A TAM-based study on museum cultural creative products consumption in China

**DOI:** 10.1371/journal.pone.0346678

**Published:** 2026-04-06

**Authors:** Changping Hu, Pengxiang Niu, Xiaoyi Chen, Ruiying Kuang, Pengxiang Cheng, Xiaojun Jiang, Rongfa Li

The authors are listed out of order. Please view the correct author order, affiliations, and citation here:

Changping Hu¹, Pengxiang Niu¹, Xiaoyi Chen¹, Ruiying Kuang², Pengxiang Cheng³, Xiaojun Jiang^4^, Rongfa Li^5^

**1** Art Institute, Xiangtan University, Xiangtan, China, **2** College of Art and Design, Changsha Institute of Technology, Changsha, China, **3** Xi’an Academy of Drama, Xi’an, China, **4** School of Public Administration, Xiangtan University, Xiangtan, China, **5** Jiangxi Institute of Fashion Technology, Nanchang, China

Hu C, Niu P, Chen X, Kuang R, Cheng P, Jiang X, et al. (2026) Does cultural identity catalyze behavior? A TAM-based study on museum cultural creative products consumption in China. PLoS One 21(2): e0341346. https://doi.org/10.1371/journal.pone.0341346
