# Supplementary material for: Does cultural identity catalyze behavior? A TAM-based study on museum cultural creative products consumption in China
Source: PLoS One. 2026 Feb 5;21(2):e0341346. doi: 10.1371/journal.pone.0341346 (PMC12875587; doi:10.1371/journal.pone.0341346)
Supplement: Attachment — Submitted filename: Plos one_Response to Review Comments and Revised Manuscript.docx [file pone.0341346.s002.docx]

****Response to Review Comments and Revised Manuscript Submission****

****– Manuscript ID [PONE-D-25-18872]****

Dear Dr. Zhuang, Prof. Abdallah Amro and Reviewers,

We would like to express our sincere gratitude to you for your time, effort, and constructive feedback on our manuscript titled “Cultural Identity as a Behavioral Catalyst: A TAM-Based SEM Study on Museum Cultural Creative Products Consumption in China” (Manuscript ID: PONE-D-25-18872). We fully appreciate the detailed and insightful comments provided by both reviewers, which have significantly helped us improve the quality and rigor of our research. Our research team has carefully reviewed and thoroughly addressed each of the concerns and suggestions raised. We have made substantial revisions to the manuscript to meet the high standards expected by PLOS ONE.

Below is a summary of how we have responded to the reviewers’ comments:

## **Summary of Revisions Based on Reviewers' Feedback**

### ****Reviewer #1: Major Concerns and Our Revisions****

1. ****Clarification of Research Contribution and Objectives****

•We have rewritten the introduction and abstract to clearly state the research gap, objectives, and contributions of the study.

•The abstract now includes key quantitative findings and highlights the theoretical and practical implications.

2.****Methodological Justification****

•We have added a detailed rationale for choosing the TAM model and its relevance to museum cultural product consumption.

•We also clarified the theoretical foundation of the proposed Cultural Identity Model (CIM).

3.****Statistical Analysis and Model Fit****

•We have elaborated on the model fit indices and clarified why certain metrics (e.g., R², f², Q²) are not applicable in our covariance-based SEM approach.

•We have included additional fit indices and path coefficients to enhance the robustness of our analysis.

4.****Ethical Considerations****

•We have added a full section on ethical approval, including parental consent procedures for participants under 18.

•We have also clarified the relevance of income data in the context of cultural consumption behavior.

5.****Discussion and Implications****

•We have expanded the discussion section to include comparisons with recent studies and provide both theoretical and practical implications.

•We have also addressed the limitations of the study and outlined future research directions.

### ****Reviewer #2: Major Concerns and Our Revisions****

1. ****Title and Consistency****

•We have revised the title to better reflect the findings and theoretical focus: “Does Cultural Identity Catalyze Behavior? A TAM-Based Study on Museum Cultural Creative Products Consumption in China”.

•We have clarified the conceptual link between TAM and cultural identity throughout the manuscript.

2. ****Readability and Language****

•We have revised the entire manuscript for clarity, grammar, and sentence structure.

•Long sentences have been simplified, and translation-style expressions have been improved.

3. ****Sample and Variable Design****

•We have added a discussion on the limitations of our sample and variable design.

•We have also suggested ways to improve representativeness in future research.

4. ****Interpretation of Insignificant Results****

•We have expanded the discussion on the insignificance of cultural identity variables, linking them to the characteristics of current cultural consumption in China.

5. ****Policy and Practical Implications****

•We have added a new section discussing how our findings can inform museum design, marketing strategies, and cultural policy development.

We sincerely hope that the revised manuscript addresses all the concerns raised and meets the publication standards of PLOS ONE. We would be very grateful if you would consider our revised submission for publication.

Thank you once again for your valuable feedback and support.

With kind regards,

**Changping Hu**

On behalf of all authors

**Art Institute of Xiangtan University**

**hcp0708@xtu.edu.cn**
