# Supplementary material for: Does cultural identity catalyze behavior? A TAM-based study on museum cultural creative products consumption in China
Source: PLoS One. 2026 Feb 5;21(2):e0341346. doi: 10.1371/journal.pone.0341346 (PMC12875587; doi:10.1371/journal.pone.0341346)
Supplement: Attachment — Submitted filename: PONE-Response to Reviewer Comments.docx [file pone.0341346.s003.docx]

### ****Response to Reviewer Comments****

****Manuscript ID:**** PONE-D-25-38750
****Title:**** Does Cultural Identity Catalyze Behavior? A TAM-Based Study on Museum Cultural Creative Products Consumption in China

#### ****General Response****

We sincerely appreciate the constructive feedback from both reviewers, which has significantly helped us refine the quality and clarity of our manuscript. We have carefully addressed all comments and made the necessary revisions to enhance the robustness, readability, and contribution of the study. Below is a point-by-point response to each comment, with specific changes highlighted.

### ****Response to Reviewer #1****

**1.Introduction:**

****Revisions:****

****The field of our research:**** We have expanded the introduction to explicitly outline the main sources (e.g., recent studies on museum cultural creative products [MCCPs] in China, Technology Acceptance Model [TAM] applications in consumer behavior) and situate the research within the fields of cultural economics, consumer psychology, and museum studies.

****Theoretical Gap:**** We have clarified the gap by emphasizing that while existing literature highlights cultural identity as a driver of MCCP consumption, few studies empirically test its relative importance compared to functional and aesthetic attributes, particularly using a TAM-based framework in the Chinese context.

****Paper Structure:**** A clear roadmap of the manuscript is now included: “This paper proceeds as follows: Section 2 reviews literature and develops hypotheses; Section 3 describes the methodology, including sample selection and data analysis; Section 4 presents results; Section 5 discusses findings in relation to existing literature; and Section 6 concludes with implications and limitations.”

****2. Discussion: Comparison with Other Results****

****Revisions:**** The discussion section now includes explicit comparisons with key studies. For example, we contrast our finding (functional/aesthetic attributes outweigh cultural identity) with Li et al. (2022), who emphasized cultural identity in Chinese MCCP consumption, and Zhang & Wang (2021).

3.****References: Alphabetical Order****

****Revisions:**** Thank you for your advice. We will revise the references according to the editor's requirements.

****4.Inclusion of Suggested Source****

****Revisions:**** We have incorporated Kačerauskas (2023) into the literature review, particularly in the section discussing “disinterestedness” in cultural consumption. This source enriches our analysis by providing a comparative perspective on how cultural institutions balance commercialization and cultural value, which we link to China’s MCCP market development.

### ****Response to Reviewer #2****

#### ****Overall Evaluation****：We thank the reviewer for their positive assessment of the manuscript’s structure, methodology, and contribution. We have addressed all minor revisions to enhance clarity, methodological justification, and result interpretation.

#### ****Specific Comments for Minor Revision****

****1.Clarity and Flow: Simplifying Long/Complex Sentences****

•****Revisions:**** Thank you very much for your suggestion. We have revised the whole text again. We have broken down dense sentences in the introduction and theoretical sections.

****2.Theoretical Justification: TAM Adaptation to Cultural Products****

•****Revisions:**** In the methodology section, we added justification for extending TAM to the Cultural Identity Model (CIM). Specifically, we explain: “TAM’s constructs of Perceived Usefulness (PU) and Perceived Ease of Use (PEOU) are adapted here to MCCPs by redefining ‘usefulness’ as functional utility (e.g., practicality, durability) and ‘ease of use’ as the ease of integrating products into daily life (e.g., portability, compatibility with lifestyle). This adaptation aligns with prior research (e.g., Venkatesh et al., 2016), which demonstrates TAM’s flexibility in non-technological contexts by focusing on perceived benefits and barriers to adoption.”

****3.Results Presentation: Figure 3 and Table 7****

•****Figure 3 (Path Diagram):**** The path diagram has been redrawn with higher resolution, enlarged font sizes, and clearer labels for latent variables and path coefficients.

•****Table 7:**** A new column titled “Hypothesis Supported?” has been added, with “Yes/No” indicators for H1–H5, providing an at-a-glance summary of hypothesis testing results.

****4.Discussion on Non-Significant Findings****

•****Revisions:**** We deepened the interpretation of non-significant cultural identity effects by exploring contextual factors: “The non-significance of cultural identity may reflect China’s MCCP market maturity: as products have become more commercialized, consumers prioritize utility over symbolism. Additionally, younger respondents may associate MCCPs with trendy aesthetics rather than cultural heritage, aligning with global youth consumer trends.”

****5.Limitations and Future Research****

•****Revisions:**** We also acknowledge potential limitations, such as the measure of cultural identity and suggest future research use multi-item scales for sub-dimensions .

•****Future Research:**** We added a suggestion to test the model with diverse demographics: “Future research could also include comparative studies across different regions of China to examine the impact of regional cultural differences on consumer behavior regarding museum cultural and creative products. Furthermore, cross-cultural comparative studies between Eastern and Western countries could be conducted, using diverse cultural samples to verify the impact of the cultural attributes of museum cultural and creative products on consumer behavior. This multi-level comparative study would help reveal the universality and specificity of cultural factors in the consumption of cultural and creative products.”

****6.Minor Typographical Checks****

•****Revisions:**** We sincerely appreciate the reviewers' valuable suggestions. We have dedicated considerable time and resources to revising these aspects. We believe this rigorous revision has significantly enhanced the quality of our manuscript. For example, We proofread the manuscript to split long sentences and standardized reference formatting. “The prevalent linear exhibition format, characterized by static chronological displays, fails to create lasting cultural memory or experiential value, necessitating a critical reassessment of institutional approaches to cultural transmission and identity formation (Eid, 2016).“ -----**→** ”The prevalent linear exhibition format relies on static chronological displays, yet it struggles to foster enduring cultural memory or meaningful visitor experiences. This limitation calls for a reevaluation of institutional strategies in cultural transmission and identity building (Eid, 2016).”

We believe these revisions have strengthened the manuscript’s clarity, methodological rigor, and contribution to the literature. We are grateful for the reviewers’ insights, which have helped refine our work. We hope the revised version meets PLOS ONE’s standards for publication.

**Sincerely,**
Changping Hu

2025-11-19
